# Adipose-Specific Cytokines as Modulators of Reproductive Activity

**DOI:** 10.3390/biomedicines13123067

**Published:** 2025-12-12

**Authors:** Marcelo Martinez-Barbitta, Andrea Biagini, Egidia Costanzi, Margherita Maranesi, Juan García-Díez, Cristina Saraiva, Beniamino Cenci Goga, Massimo Zerani

**Affiliations:** 1 Sistema Reproductivo Veterinario Integral Uruguay, SRVI_UY, Nueva Helvecia 70300, Uruguay; 2 Dottorato di Ricerca in Sanità e Scienze Sperimentali Veterinarie, Università di Perugia, 06121 Perugia, Italy; 3 Dottorato di Ricerca in Patologie Infiammatorie ed Infettive, Strategie Terapeutiche e Biodiritto, Università di Perugia, 06121 Perugia, Italy; andrea.biagini@dottorandi.unipg.it; 4 Dipartimento di Medicina Veterinaria, Università di Perugia, 06121 Perugia, Italy; egidia.costanzi@unipg.it (E.C.); margherita.maranesi@unipg.it (M.M.); massimo.zerani@unipg.it (M.Z.); 5 Centro de Ciência Animal e Veterinária, Universidade de Trás-os-Montes e Alto Douro, 5000-911 Vila Real, Portugal; juangarciadiez@utad.pt (J.G.-D.); crisarai@utad.pt (C.S.); 6 Laboratório Associado para a Ciência Animal e Veterinária, Universidade de Lisboa, 1300-477 Lisboa, Portugal; 7 Faculty of Veterinary Science, Department of Paraclinical Sciences, University of Pretoria, Pretoria 0110, South Africa

**Keywords:** adipokines, reproduction, white adipose tissue, adiponectin, leptin, omentin, resistin, visfatin

## Abstract

Adipose tissue is characterized by specialized lipid handling cells called adipocytes, which function as the primary energy reservoir. Like many other cell types, adipocytes have highly plastic properties, such as the conversion of white adipocytes into brown or beige adipocytes, which produce heat, and pink adipocytes into mammary cells synthesizing and secreting milk. Highly specialized adipose tissue depots are present in various species, such as male orangutans with prominent fat-filled facial flanges indicating hierarchical status, or cetaceans with the melon, a specialized adipose tissue for echolocation. Adipose tissue is now considered a true endocrine organ that regulates various physiological mechanisms through the hormonal secretion of adipokines, which modulate systemic metabolism and physiological processes. In particular, the role of adipokines in the control of the reproductive axis and their participation in the regulation of fertility have been widely reported. This review summarizes the current state of research on the effects of adipose-specific cytokines on the male and female reproductive systems.

## 1. Introduction

The mechanism of storing energy in the form of lipids is highly conserved and shared by unicellular and multicellular organisms throughout evolutionary phylogeny [1]. Unicellular organisms, both prokaryotes and eukaryotes, store lipids in intracellular organelles known as lipid droplets or lipid bodies, whereas multicellular organisms have developed specialized cells to store them [2,3,4]. These specialized cells are found in both invertebrates and vertebrates, although they may have evolved convergently by sequestration of lipids from the extracellular environment [4].

Mammals have four main types of adipocytes—white (white adipose tissue, WAT), beige, brown (brown adipose tissue, BAT) and pink—scattered throughout the body in structured and distinct deposits [5]. Lipid storage and release are the responsibility of white adipocytes, while thermogenesis is the specialization of beige and brown adipocytes, capable of expending nutritional energy in the form of heat, and pink adipocytes are the result of transdifferentiation of white adipocytes into mammary alveolar cells during gestation and lactation [5]. Highly specialized adipose stores are present in some mammalian species [1]. Male orangutans display prominent fat-filled facial flanges that develop in adulthood and are largely absent in adult females and pubescent males [6]. These flanges determine the hierarchical status, dominance, and successful reproductive fitness of estrus females. Odontocetes use the melon, an oval-shaped organ formed by adipose tissue and located in the center of the forehead above the maxilla, for echolocation [7]. Ultrasound waves generated by phonic lips travel and collimate within the melon. Upon echoing, the return sound waves pass through the fat mass present in the jaw and are then perceived by the auditory system. Elephants evolved prominent plantar adipose tissue that dissipates pressure and protects adjacent skeletal structures from mechanical shock during gait and walking [8]. In addition to its shock-absorbing function, plantar adipose tissue has been hypothesized to participate in the perception of seismic signals [9]. Behavioral studies evidenced the capacity to perceive low-frequency seismic signals, even if its mechanism remains unclear. It has been suggested that elephants can perceive ground-based sound through the sound conduction capability of foot adipose and bone [9]. The prominent use of adipose deposits modified for sound conduction in odontocetes suggests the convergent evolution of this function in the plantar adipose of elephants. The humps in camels contain adipose tissue that plays an adaptive role in the desert environment [10]. During fasting periods, camels mobilize fatty acids from hump stores. The unique positioning of the adipose tissue in the humps, away from the body core, prevents the camels from overheating [10].

White, beige, and brown adipocytes exhibit endocrine activity—unlike pink adipocytes—so much that adipose tissue is considered the largest endocrine tissue in mammals [5]. Adipose tissues secrete a large number of bioactive compounds, called adipokines, that can be divided into adipose-specific cytokines directly released by adipocytes (adiponectin, leptin, omentin, resistin, and visfatin) and non-adipose-specific cytokines (such as retinol binding protein 4, lipocalin 4, irisin, chemerin, interleukin (IL) 6, IL1β, and tumor necrosis factor α) secreted by various cell types [11]. Adipokines modulate various physiological processes, such as the regulation of energy and appetite, hematopoiesis, insulin sensitivity, osteogenesis, thermogenesis, chondrogenesis, angiogenesis, atherosclerosis, blood pressure, and neuroendocrine and immune responses [11,12,13]. The hypothalamic–pituitary–gonadal axis is profoundly affected by adipokines, highlighting the substantial impact that these bioactive compounds have on reproductive function [14].

The aim of this review is to focus on specific adipokines produced by adipocytes and their effect on the reproductive physiology of male and female mammals.

## 2. Adiponectin

Adiponectin (APN) is the most abundant circulating adipokine secreted by WAT [15,16]. Its plasma levels show an inverse correlation with the degree of obesity and the amount of adipose tissue [17]. APN consists of approximately 240 amino acids and has a molecular weight of approximately 25 kDa, depending on the mammalian species considered [18]. APN is encoded by the APN gene [19] and shows three oligomeric complexes [20]: APN-LMW (low-molecular-weight trimer), APN-MMW (medium-molecular-weight hexamer), and APN-HMW (high-molecular-weight multimer). Three different receptors are available for APN; two (AdipoR1 and -R2) were initially identified [21], and later a third nonsignaling receptor for adiponectin, T-cadherin, was recognized [22]. The different forms of adiponectin bind to specific receptors: trimers bind to AdipoR1, hexamers bind to AdipoR2, and hexamers and high molecular weight multimers bind to T-cadherin [21,22]. APN triggers various signaling cascades, which involve phosphorylation of AMP-activated protein kinase (AMPK) and p38 mitogen-activated protein kinase (p38 MAPK), peroxisome-proliferator-activated receptor (PPAR) α and γ, PPARγ-coactivator-1α (PGC-1α) and insulin receptor substrates 1 (IRS-1) [23,24]. Adiponectin receptors have been identified in the hypothalamus, including the paraventricular nucleus and periventricular areas, and particularly in hypothalamic GnRH neuron cells [23]. Adiponectin inhibits the transcription of the kisspeptin (KISS) 1 gene and GnRH secretion [25,26]. Kiezun et al. [27] reported that APN treatment increases FSH release in primary pituitary cells (Table 1).

In contrast, other authors found that APN reduces LH secretion and GnRH-induced LH release in pituitary cell cultures [28,29]. Adiponectin receptors have been identified in gonadotropin-producing cells in the pars distalis but not in the pars tuberalis of the pituitary [30]. Restricted feeding in prepubertal ewes increases serum adiponectin but alters the hypothalamic–pituitary–ovarian axis by downregulating GnRH, LHβ, and FSHβ production [31].

### 2.1. Male

The expression of genes encoding AdipoR1 and -R2 was detected in the rat testes, with developmental changes and gonadotropin regulation observed for AdipoR2 mRNA, and prominent levels of AdipoR1 found in seminiferous tubules [42]. Furthermore, recombinant adiponectin significantly inhibited basal and human chorionic gonadotropin-stimulated testosterone secretion ex vivo [42] (Table 2).

On the contrary, in the MA-10 mouse Leydig cell line, APN treatment enhances progesterone production by increasing the expression of cholesterol carrier steroidogenic acute regulatory protein (StAR) and the cytochrome P450 (CYP) 11A1 enzyme, suggesting that high APN levels may induce testosterone production from Leydig cells [43]. In ovine species, AdipoRs expression has been demonstrated in the male reproductive tract [60] and in spermatozoa [44]. This latest research showed that certain indices of sperm motility, including curvilinear speed, rectilinear speed, mean walking speed, linearity, oscillation and straightness, were significantly correlated with AdipoR1 expression [44]. Regarding the parameters of the sperm, adiponectin was positively associated with the concentration of the sperm, the total sperm count, and the percentage of spermatozoa with normal morphology [45]. Recently, the presence, location, and gene expression of AdipoR1 in reproductive tissues of the male rams were reported, during their nonbreeding season [46]. Immunohistochemistry showed the presence of AdipoR1 in all glandular and germline epithelial cells. In testicles, epididymis, vas deferens, bulbourethral glands, seminal vesicles, and prostate, AdipoR1 transcription has been observed, with higher levels in the prostate. This study highlights the role of the APN/AdipoR1 system in the regulation of testicular activity in males during the nonreproductive season [46].

### 2.2. Female

In pigs, adiponectin stimulates genes involved in ovarian follicle remodeling, such as cyclooxygenase (COX) 2, prostaglandin (PG) E synthase, and vascular endothelial growth factor (VEGF), in granulosa cells [61] (Table 3).

In bovine granulosa cells, adiponectin mRNA levels increase 25 h after GnRH treatment, while AdipoR2 mRNA is upregulated at 20, 25, and 60 h after GnRH [62]. In porcine luteal cells, adiponectin modulates genes involved in steroidogenesis, PG synthesis, and vascularization, thus influencing corpus luteum (CL) growth and function [63]. Adiponectin levels are also elevated during the mid and late luteal phases, indicating its potential involvement in sustaining CL activity [79]. Furthermore, adiponectin improves embryo development to the blastocyst stage in vitro [64] and affects both PG synthesis and VEGF expression in endometrial cells during pregnancy [65]. Both adiponectin and insulin modulate the steroidogenic enzymes StAR and CYP19A3 in endometrium and myometrium during pregnancy and the estrous cycle [66,67]. In sheep, maternal obesity alters the expression of fetal adiponectin, affecting adipogenesis and insulin sensitivity [83]. Increased maternal nutrition stimulates the expression of fetal leptin and adiponectin mRNA in perirenal fat depots [84,85]. During early lactation in dairy cows, adiponectin protein levels in adipose tissue are reduced at one week postpartum compared to five months of gestation, suggesting altered energy metabolism during this period [80].

## 3. Leptin

Leptin consists of 167 amino acids (16 kDa), encoded by a gene called obese (OB) in mice or leptin (Lep) in humans [15,86]. Leptin is a cytokine of the IL-6 family, which expresses its pleiotropic characteristic and action in various tissues through the leptin receptor (LEPR). This receptor belongs to the class I cytokine superfamily and exists in six isoforms (a–f) due to alternative RNA splicing [11]. LEPRb is the only isoform containing a full-length intracellular domain, enabling full activation of Janus kinase (JAK) 2 as well as the signal transducer and activator of transcription proteins (STAT) 3 and 5 pathways [87]. Leptin affects all components of the hypothalamic–pituitary–gonadal axis; it demonstrates stimulatory effects in the central nervous system and consequently indirectly modulates gonadic function through GnRH [32]. Although leptin stimulates GnRH secretion, GnRH neurons do not express LEPR [33], suggesting that leptin effects are mediated through neuropeptides, including kisspeptin [32], with leptin [88]. The processes modulating GnRH neurons—and potentially disrupting their pulsatile release—depend on the energy status and circulating leptin levels [14,34,35]. Pituitary gland cells express LEPR and respond to changes in leptin concentrations; in fact, leptin induces the expression of activin mRNA in gonadotropes, crucial for the synthesis of FSH [36,37]. The onset of puberty is defined by LH nocturnal pulses. In this context, Suter et al. [38] found that nocturnal leptin levels are significantly higher before the onset of puberty, such as those of growth hormone (GH) and insulin-like growth factor (IGF) 1, in monkey males. These authors [38] suggested that the GH–IGF-1 axis, and in turn GnRH and LH, is stimulated by leptin. In sheep, maternal undernutrition during pregnancy increases fetal hypothalamic leptin receptor expression, which normalizes with improved maternal nutrition later in gestation [89]. On the contrary, maternal obesity in sheep reduces leptin signaling in the pituitary and alters GH/IGF-1 regulation [90].

### 3.1. Male

The blood–testis barrier (BTB) can be crossed by leptin, resulting in its presence in testicular fluid and seminal plasma [91]. Leptin receptors are found on spermatozoa, germ cells, somatic cells, epididymis, Leydig cells, Sertoli cells and epithelial cells of seminal vesicles and prostate [47,48]. Leptin modulates testosterone production in Leydig cells and androgen-binding protein, testicular fluid, inhibin, activin, and factors necessary for spermatogenesis in Sertoli cells [49,50]. Leptin reduces oxidative stress and sperm apoptosis and positively influences mitochondrial function and energy sources [51]. Its absence or low concentration leads to decreased steroid hormones, increased germ cell apoptosis and expression of pro-apoptotic genes, and vacuolization in Sertoli cells [52,53,54]. In male ob/ob mice, the absence of leptin results in infertility, which can be restored with leptin therapy [92]. However, when leptin concentration is very high, apoptosis rates increase in all testicular cell types and abnormal sperm numbers increase, while motility and sperm concentration decrease, and BTB is disrupted [53,92,93]. Alterations in steroidogenic enzyme pathways and enzymes involved in sperm activity have been demonstrated in leptin-deficient mouse testes [52]. Furthermore, leptin deficiency leads to impaired spermatogenesis, increased germ cell apoptosis, and overexpression of pro-apoptotic genes within the testes [94].

### 3.2. Female

In pigs, leptin modulates estradiol secretion in vitro [68]. In sheep, food deprivation and, consequently, low leptin levels reduce oocyte quality [69,70]. During the luteal phase in pigs, leptin exhibits antiapoptotic properties by suppressing caspase-3 activity and counteracting the effects of IGF-I to maintain appropriate cell numbers in CL [71]. Leptin also promotes the nuclear and cytoplasmic maturation of oocytes by activating the MAPK pathway, increasing maturation to metaphase II, cyclin B1 expression, and embryo development [72,73]. Leptin protects porcine oocytes from high-glucose damage, enhancing glycolysis and maturation [74]. In sheep, leptin added to culture medium increases antrum formation, follicular growth, and the proportion of oocytes reaching metaphase II [75]. In sheep, fat mass regulates plasma leptin levels, although these can change during pregnancy [95,96,97].

## 4. Omentin

Omentin shows two homologous forms (omentin-1 and omentin-2), encoded by two adjacent genes [98]. Omentin-1, also called endothelial lectin HL-1, intelectin-1, or intestinal lactoferrin receptor, is composed of 313 amino acids and (35 kDa) [13,99]. Omentin-1 activates the c-Jun N-terminal kinase (JNK) via AMPK/endothelial nitric oxide synthase/nitric oxide and potentially blocks the extracellular signal-regulated kinase (ERK)/nuclear factor κ-light-chain-enhancer (NF-κ) B pathway [100,101]. Omentin-1 shows direct effects on hypothalamic neurons; even if the effects on GnRH release have not yet been proven [39]. A recent study [102] reported that the expression of omentin-1 depends on the concentrations of LH, FSH and GnRH, in the porcine anterior pituitary gland.

### 4.1. Male

The role of omentin-1 in the cellular and molecular mechanisms of the male reproductive system remains unclear [103]. Recent studies have localized omentin-1 in human sperm and male reproductive tissues [55,103]. Sperm omentin-1 has been reported to originate from seminal vesicles and that inflammatory conditions increase its levels, which negatively correlate with sperm parameters [55].

### 4.2. Female

Omentin-1 modulates the expression of mRNA of other adipokines and their receptors in porcine granulosa cells, reducing apelin levels and increasing those of leptin, while vaspin is unaffected [76]. These findings suggest that omentin-1 acts as an auto and paracrine regulator within the ovarian microenvironment in different pig breeds [76]. Elevated expression of omentin-1 was observed in ovarian follicles, with follicular fluid concentrations higher in Large White Pigs than in Meishan pigs [104]. In both breeds, omentin-1 levels increased throughout the estrous cycle. However, gonadotropins and steroids increased omentin-1 levels in both granulosa and theca cells of Large White pigs, while only LH and testosterone stimulated omentin-1 in Meishan pigs ERK1/2 and phosphoinositide 3-kinases signaling pathways [104]. In sheep, omentin-1 was identified in ovarian tissues [83]. In dairy cows, serum omentin-1 concentrations are significantly higher at delivery compared to pre- and post-parturition [77]. Omentin-1 was positively correlated with plasma glucose, non-esterified fatty acids, and β-hydroxybutyrate, and negatively with triglycerides [77]. These findings indicate that omentin-1 may play a role in energy metabolism during the peripartum period, especially around conception, when fetal energy demands peak [77].

## 5. Resistin

Resistin is a polypeptide of 108 amino acids, with a molecular weight of 12.5 kDa and a cysteine-rich structure, encoded by the resistin gene on chromosome 19 [105]. Toll-like receptor (TLR) 4 and adenylate-cyclase-associated protein (CAP) 1 have been identified as main resistin receptors, with subsequent activation of the JNK and p38 MAPK pathways [106]. Maillard et al. [107] reported that resistin also acts via the AMPK and ERK1/2 signaling pathways. Resistin increases the B-cell lymphoma (Bcl)-2-associated X protein (BAX)/Bcl-2 ratio and activates the MAPK3/1, protein kinase B (PKB), and STAT3 pathways [78]. Resistin inhibits LH secretion in mice, and its impact on pituitary cells appears to be concentration-dependent [40].

### 5.1. Male

In Leydig cells, resistin decreases STAR expression and, consequently, steroidogenesis by negatively modulating the AMPK pathway [56]. Wagner et al. [57] reported that Sertoli cells exposed to high resistin levels interrupt maturation, remain at prepubertal quiescent state, and negatively affecting spermatogenesis initiation and maintenance of spermatogenesis. Resistin adversely affects sperm vitality and morphology, but not basic sperm parameters [45]. Additionally, an inverse correlation has been reported between resistin levels in seminal fluid and sperm motility and vitality [58].

### 5.2. Female

Resistin improves porcine luteal cell viability by inducing autophagy, supporting CL function [78]. In bovine ovaries, resistin is abundant during CL regression and early pregnancy, with receptor expression suggesting local involvement in CL regulation [79]. In dairy cows during early lactation, plasma resistin concentration is lower before calving, peaks at one week postpartum and gradually declines to pre-calving levels by six weeks postpartum [80]. Weber et al. [81] similarly noted resistin concentrations rising toward pregnancy and falling to pre-calving levels within one week after calving. Resistin mRNA and protein levels are higher at one week post-partum, compared to five months of gestation [80]. Plasma resistin is positively correlated with non-esterified fatty acids and negatively with milk yield, dry matter intake, and energy balance [80]. Recombinant bovine resistin promoted glycerol release and stimulated adipose triglyceride lipase and hormone-sensitive lipase gene expression, increasing lipid mobilization [80]. Early lactation also reduces the phosphorylation of the insulin receptor β subunit, insulin receptor substrates (IRS) 1, IRS-2, PKB, and MAPK/ERK1/2, decreasing insulin sensitivity [80].

## 6. Visfatin

Visfatin is a protein of 491 amino acids with a molecular weight of 52 kDa, acting also as an intracellular enzyme, hence the second name nicotinamide phosphoribosyltransferase (NAMPT) [108,109]. Intracellular visfatin facilitates NAD^+^ biosynthesis, promoting lipid storage [86,110,111]. The extracellular isoform acts as a classical hormone with endocrine, paracrine, and autocrine actions [112,113]. Specific receptors for visfatin have not yet been identified, although visfatin shows affinity for insulin and TLR-4 receptors [25,114,115] and regulates ERK1/2, JAK2/STAT3, AMPK, and IκB kinase/NF-κB signaling [112,113,116]. Visfatin modulates the hypothalamic–pituitary–gonadal axis, influencing the secretion of GnRH from hypothalamus and LH from the pituitary [16]. High visfatin levels reduce KISS-1 hypothalamic neuron mRNA, suggesting that this adipokine negatively regulates GnRH and LH secretion through downregulation of the KISS-1 system [41].

### 6.1. Male

The role of visfatin in male fertility has not been well studied [117,118]. Visfatin induces testosterone secretion by cultured Leydig cells through Ras 1 kinase [59], while other studies report a negative correlation between visfatin and sperm parameters [41]. Visfatin is present in Sertoli and Leydig cells, as in well as spermatozoa [119,120]; in particular, decreased expression in Leydig cells results in lower serum testosterone levels [121].

### 6.2. Female

Visfatin expression in porcine ovarian follicles is upregulated by LH, FSH, estradiol and progesterone, downregulated by insulin, and modulated dose-dependently by PGE2 and PG2α [113]. In luteal cells, visfatin is increased by progesterone and decreased by PGs, with LH and insulin effects depending on cycle phase [113]. In bovine granulosa cells, visfatin mRNA expression is upregulated twenty hours after GnRH stimulation [62]. Visfatin supports the steroidogenesis and proliferation of bovine granulosa cells: when used alone or with IGF1, it increases estradiol and progesterone secretion and expression of StAR and 3β-hydroxysteroid dehydrogenase, promoting follicular function and oocyte maturation [82].

## 7. Conclusions and Future Directions

**Figure 1:**
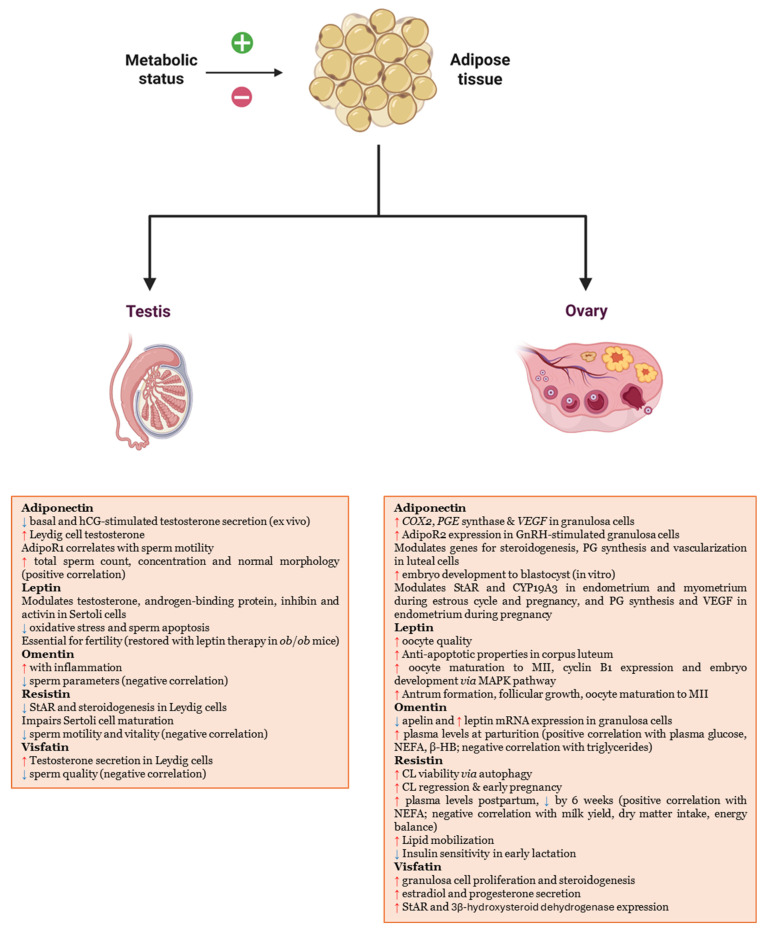
Effect of specific-adipose adipokines on male and female gonads.

The discovery of the first adipose-specific cytokine, leptin, revealed that white adipose tissue (WAT) plays an important endocrine role, affecting systemic regulation of various organs and tissues—including those of reproductive interest—through the actions of subsequently identified adipokines. Metabolic status, reflected by changes in WAT and specific adipokine secretion profiles, impacts the morphophysiology of both male and female reproductive systems (Figure 1).

The complex interaction between adipokines and human reproductive functions has attracted considerable attention in recent years [122]: in women, body fat influences various reproductive parameters, including menstrual cycles, fertility, and pregnancy outcomes [14], while leptin and adiponectin determine ovarian reserve [123]; in males, a Mendelian randomization study [124] highlights how lipidome impacts male fertility.

This review focuses on adipose-specific cytokines, but the topic of adipokines is much broader, with studies meriting a review for each subject covered. Among the various examples, the link between obesity, adipokines, and cancer, especially breast cancer, is among the most studied. There is strong evidence linking increased adiposity with the development of 13 types of cancer, with important direct crosstalk between adipose tissue and various organs [125]. The connection between breast cancer and obesity depends on complex and interconnected biological mechanisms, including dysregulation of adipokines, which together generate a pro-tumorigenic environment, thus favoring the initiation, progression and recurrence of cancer [126]. Women with obesity have a higher chance of developing breast cancer and a greater risk of exacerbating the disease [127]. Adipokines have a significant impact on this link, especially leptin and adiponectin [128]. Leptin behaves as a growth factor in breast cancer, activating multiple oncogenic signaling pathways [129] and improving estrogen synthesis in adipose stromal cells [130]. Adiponectin is an anti-inflammatory factor with antiproliferative properties; in particular, it suppresses the proliferation of breast cancer cells, induces apoptosis, and alters metastatic potential [126]. Adiponectin counteracts leptin protumor effects, suggesting that obesity-associated breast carcinogenesis is mainly regulated by a leptin–adiponectin axis imbalance [126]. Recently, omentin-1 was suggested as a potential tumor suppressor, as it was found to be inversely related to abdominal fat, which is associated with a higher risk of breast cancer [131]. This relationship between omentin-1 and breast cancer has been proposed very recently, so more conclusive studies are needed [127].

From the large number of studies on the endocrine activity of adipose tissue and its effects on reproductive activity, two possible new fields of research emerge: brown adipose tissue and the gut microbiota. Brown adipose tissue is a metabolically active tissue that secretes a complex array of adipokines, called batokines [132], such as cytokines, factors, proteins, metabokines, and extracellular vescicles that signal and mediate different metabolic effects in target organs, including white adipose tissue [133], suggesting a possible effect on reproductive activity. Significant alterations in the composition and diversity of the gut microbiota, a condition known as dysbiosis, are associated with obesity [134]. The gut microbiota is a key environmental factor in the predisposition to adiposity, since it can regulate body fat storage, adipocyte metabolism, and inflammation of adipose tissue inflammation [135]. This evidence also suggests possible effects on reproductive activity, which will have to be demonstrated in future studies.

In conclusion, adipokines emerge as promising therapeutic targets that could clarify the mechanisms underlying fertility problems associated with altered nutritional status, in both human and veterinary contexts. Understanding the relationship between metabolic state, represented by adipose tissue and its adipokines, and reproductive dysfunctions can contribute to the development of new strategies for the treatment of male and female infertility.

## Figures and Tables

**Table 1:** Effects of adipose-specific cytokines on the hypothalamic–pituitary axis.

Adipokines	Effects
Adiponectin	AdipoRs present in the hypothalamus, particularly in the hypothalamic GnRH neuron cells [23].
	Inhibition of KISS 1 gene transcription and GnRH secretion [25,26].
	Increase in FSH release in primary pituitary cells [27].
	Reduction in LH secretion and GnRH-induced LH release in pituitary cell cultures [28,29].
	AdipoRs present in gonadotropin-producing cells [30]. Restricted feeding increases serum adiponectin and downregulates GnRH, LHβ, and FSHβ production [31].
Leptin	Stimulation of GnRH secretion, mediated by KISS [32,33].
	Energy status and circulating leptin levels modulate GnRH neurons with disrupting GnRH pulsatile release [14,34,35].
	LEPRs present in pituitary cells [36].
	Increase in mRNA expression for activin in gonadotropes [37].
	Stimulation of GH–IGF-1 axis and, in turn, of GnRH and LH release [38].
Omentin	Direct action on hypothalamic neurons; however, the effect on GnRH release remains unknown [39].
Resistin	Inhibitory effect on LH secretion [40].
Visfatin	Effects on GnRH secretion from the hypothalamus and LH from the pituitary [16].
	Reduction in hypothalamic KISS-1 mRNA expression [41].

**Table 2:** Effects of adipose-specific cytokines on male gonads.

Adipokines	Effects
Adiponectin	Inhibition of basal and human choriogonadotropin-stimulated testosterone secretion Ex Vivo [42].
	Induction of testosterone production from the Leydig cells [43].
	Indices of sperm motility significantly correlated with the expression of AdipoR1 [44].
	Positive association with sperm concentration, total sperm count, and percentage of spermatozoa with normal morphology [45].
	AdipoR1 presence and location and its gene expression in the reproductive tissues of the male ram, during its nonbreeding season [46].
Leptin	Receptors in spermatozoa, germ cells, somatic cells, epididymis-mis, Leydig cells, Sertoli cells and epithelial cells of seminal vesicles and prostate [47,48].
	Modulation of testosterone production in Leydig cells and androgen-binding protein, testicular fluid, inhibin, activin in Sertoli cells [49,50].
	Reduction in oxidative stress and sperm apoptosis [51].
	In male ob/ob mice, the absence of leptin leads to a lack of fertility, which is restored with leptin therapy [52,53,54].
Omentin	Inflammatory conditions increase its levels, while they are negatively correlated with sperm parameters [55].
Resistin	In Leydig cells, decrease in STAR expression and steroidogenesis [56].
	In Sertoli cells, interruption of maturation and maintenance of the prepubertal quiescent state [57].
	Negative correlation with sperm motility and vitality [58].
Visfatin	In cultured Leydig cells, induction of testosterone secretion [59].
	Negative correlation with sperm parameters [41].

**Table 3:** Effects of adipose-specific cytokines on female reproductive tissues.

Adipokines	Effects
Adiponectin	Stimulation of genes for COX2, PGE synthase, and VEGF, in granulosa cells [61].
	Upregulation of AdipoR2 in GnRH-treated granulosa cells [62].
	Modulation of genes for steroidogenesis, PG synthesis, and vascularization in luteal cells [63].
	In vitro improvement of embryo development to the blastocyst stage [64].
	Affects PG synthesis and VEGF expression in endometrial cells during pregnancy [65].
	Modulation of StAR and CYP19A3 in the endometrium and myometrium during pregnancy and estrous cycle [66,67].
Leptin	Modulation of estradiol secretion in vitro [68]. Food deprivation, which determines low levels of leptin, reduces oocyte quality [69,70].
	Antiapoptotic properties by suppressing caspase-3 activity and counteracting IGF-I effects in CL [71].
	Activation of the MAPK pathway, increase in oocyte maturation to metaphase II stage, expression of cyclin B1 and embryo development [72,73].
	Protection of oocytes from high-glucose-level damage, enhancing glycolysis and maturation [74].
	Increase in antrum formation, follicular growth, and the proportion of oocytes reaching metaphase II [75].
Omentin	Modulation of mRNA expression for adipokines and their receptors in granulosa cells, reducing apelin levels and increasing those of leptin [76].
	Levels were significantly higher at delivery compared to the pre- and post-parturition and positively correlated with plasma glucose, non-esterified fatty acids and β-hydroxybutyrate, and negatively with triglycerides [77].
Resistin	Enhances porcine luteal cell viability through autophagy, supporting CL function [78] Local involvement in CL regulation [79]. Oscillating expression related to pregnancy [80,81]. Positively correlated with NEFA; negatively with milk yield, DMI, and energy balance [80]. Recombinant resistin promotes lipid mobilization [80]. Early lactation reduces insulin sensitivity via reduction in IRβ, IRS-1/2, PKB, and MAPK/ERK1/2 phosphorylation [80].
Visfatin	Supports granulosa cell steroidogenesis and proliferation [82].
	Alone or with IGF1 increases estradiol and progesterone, promoting follicular function and oocyte maturation [82].

## Data Availability

Not applicable.

## Abbreviations

The following abbreviations are used in this manuscript:

AdipoR	Adiponectin receptor
AMPK	AMP-activated protein kinase
APN	Adiponectin
BAT	Brown adipose tissue
BTB	Blood-testicular barrier
CAP1	Adenylate-cyclase-associated protein 1
CL	Corpus luteum
COX	Cyclooxygenase
CYP	Cytochrome P450
ERK	Extracellular Signal-Regulated Kinase
GH	Growth hormone
HMW	High-molecular-weight
IGF	Insulin-like growth factor
IL	Interleukin
IRS	Insulin Receptor Substrates
IRβ	Insulin Receptor β subunit
ITLN	Intelectin
JAK	Janus kinase
JNK	c-Jun N-terminal kinase
KISS	Kisspeptin
LEPR	Leptin Receptor
LMW	Low-molecular-weight
MAPK	Mitogen-Activated Protein Kinase
MMW	Medium-molecular-weight
NAMPT	Nicotinamide Phosphoribosyltransferase
NF-κB	Nuclear Factor kappa-light-chain-enhancer
PG	Prostaglandin
PKB	Protein Kinase B
PPAR	Peroxisome-proliferator-activated receptor
StAR	Steroidogenic Acute Regulatory
STAT	Signal Transducer and Activator of Transcription
TLR	Toll-like receptor
VEGF	Vascular Endothelial Growth Factor
WAT	White adipose tissue
